# The Healthcare Staffs’ Perception of Parents’ Participation in Critical Incidents at the PICU, a Qualitative Study

**DOI:** 10.3390/nursrep11030064

**Published:** 2021-08-30

**Authors:** Julia Hansson, Amanda Hörnfeldt, Gunilla Björling, Janet Mattsson

**Affiliations:** 1 Children’s Perioperative Medicine and Intensive Care, Karolinska University Hospital, 17164 Stockholm, Sweden; julia.hansson@sll.se (J.H.); amanda.hornfeldt@sll.se (A.H.); 2 Department of Health Sciences, The Swedish Red Cross University College, 12141 Stockholm, Sweden; gunilla.bjorling@rkh.se; 3 Kilimanjaro Christian Medical University College Moshi, Tumanini University, Dar es Salaam 2240, Tanzania; 4 Department of Neurobiology, Care Sciences and Society, Karolinska Institutet, 14183 Stockholm, Sweden; 5 Department of Learning, Informatics, Management and Ethics, Karolinska Institutet, 17165 Stockholm, Sweden

**Keywords:** children intensive care, parental presence, nursing, power relations

## Abstract

Background: Internationally, there are very few guidelines regarding how near relations can be taken care of on a children’s intensive care unit. Despite knowledge about the positive effects of parental presence, staff frequently reject parents out of insecurity. This study aimed to investigate health professionals’ understanding of letting parents be present throughout critical situations. A qualitative method with semi-structured interviews was used to answer the aim of his study. Nine persons participated in the study, both physicians and nurses. The result showed that health professionals’ main view is that parents’ presence is positive. However, their presence often has lower priority than the medical focus of the child and the health professionals’ concern of failure. Conclusion: Health professionals have the power to decide if parents can be present in critical situations. Only when a parent demands to be present does that demand beat the decisions made by health professionals. Lack of resources within the team and fear of parents becoming a disturbance or a distraction are cited as the primary reasons not to let parents be present.

## 1. Introduction

When children are admitted to the Pediatric Intensive Care Unit (PICU), parents are often not present in critical situations due to a lack of guidelines and organizational shortcomings. However, parents should be present in critical situations as it reduces stress and uncertainty [1] and is beneficial for children and parents [2]. Nevertheless, medical staff is often hesitant to let parents be present at critical incidents in the PICU. The reasons why are largely unknown. Research suggests that this hesitance builds on a misguided perception that the presence of parents could create stress and anxiety [3,4,5]. When parents instead are given a possibility to participate, they may give quick access to the child’s health history [6,7]. Therefore, we addressed the health care staff’s perception of parents’ participation in critical situations.

Children’s need to have parents present is equally essential worldwide, whether in a critical situation or during an everyday chore [8,9,10]. In a PICU, it is vital to make sure that the child and the parents are allowed to utilize the child’s resources for recovery and experience of well-being and health [10]. The parents are also one of the best resources to uncover what the child is trying to communicate [11]. The child’s best interests must be a primary consideration and grant the child empathy and integrity. However, a child’s best interests must be decided in each case [12]. Nursing care emerging from a child-centered perspective acknowledges the child as a person, respects the child’s need for space and information and their own decisions [13], allowing the child to be at the center of their care. The focus should also be on seeing the child as part of a family [14], enabling children to have a parent or other close relative with them whenever they want [12,15]. Nurses tend to be more likely to involve parents than physicians [3,6,8].

The increased anxiety parents may feel and the fear of what their child is going through is described as reasons to exclude them [4,6]. There is also a belief that parents could influence medical decisions [4]. Alternatively, there is not enough staff, space, or time to take care of them [7]. Lack of experience in similar situations is also described as a reason for exclusion [3,4]. After experiencing a critical situation with parents present, nurses’ and physicians’ confidence grows, and anxiety decreases, and they become more likely to offer parents to participate [5,6]. Allowing parents to participate while experiencing that nothing vital to the child was missing eased the nurses’ conscience [6]. Healthcare staff describes that guidelines for including parents in critical situations would facilitate decision-making, but guidelines are missing in many workplaces [7]. Internationally, three countries have guidelines regarding parents’ participation in resuscitation: United States, Canada, and the United Kingdom [8].

Parents view the PICU stay as chaotic and unrealistic [16] (a parent is defined as a person who cares for or supports a close relative, here the parents of a child [15]). They describe stress, fear, and anxiety about seeing their child in the PICU and emotional trauma remaining after discharge from the PICU [17]. If the health care staff provides clear and timely information, parents feel seen and recognized as parents [18]. However, disseminating information must be adapted to the situation to involve parents and create security and peace for their children [17]. Parents believe that their presence will increase the chances of survival of their child and reduce the child’s anxiety and fear. If they are not allowed to participate in critical situations, continuous information is pivotal [16,19]. Parents want their child to be met holistically, as a human being through physical, mental, existential, and spiritual dimensions [20].

There is a particular concern about separating the child from a parent if the child does not survive [19]. The underlying assumption is that if the family sees that the health care staff are doing everything to save the child, their fear and nervousness reduce, and if the child passes away, the family can say goodbye [6]. From the parents’ perspective, if they participated in resuscitation, anxiety and depression decrease, and they perceive the grieving process as more tolerable [21]. It is pivotal that parents are allowed to participate in critical situations. However, healthcare staff’s attitude to involving parents in critical situations seems to be the threshold for such an endeavor. Therefore, we decided to explore how health care staff perceive parents’ participation in critical situations.

### Aim of the Study

This study aimed to explore the health care staff’s perception of parents’ participation in critical situations.

## 2. Materials and Method

In this study, a qualitative design was chosen to understand a whole and gain a deeper understanding of the phenomena. The method is considered flexible, and by collecting data where the phenomena have their natural habitat, the phenomena are more relevant to study [22]. A qualitative method with semi-structured interviews was deemed the best way to answer the aim of the study.

The authors have followed COREQ reporting guidelines. The material was analyzed using qualitative content analysis with an inductive approach [22]. A method based on inductive data moves from the specific to the general, so a larger whole or a general statement is reached [23].

### 2.1. Selection

A strategic selection, inviting persons with diverse clinical experiences and diverse experience of critical incidents, was applied to gain as much variation as possible regarding experiences from critical incidents, experiences, and responsibilities in their professional practice, which according to Polit and Beck [22] gives depth to the result. Five nurses and four physicians participated in the study. All participants came from the same department but had work-based experiences from diverse departments. The nurses who participated in the study had worked in the department as intensive care nurses between 10 months and 22 years. The physicians’ experience in the ward varied between four months and eighteen years. Among both nurses and physicians, there was a mixed background from both adult and pediatric care. Inclusion criteria for participating in the study were that the informant should be a licensed physician or registered nurse with at least one previously experienced critical situation. The informant should work in a child intensive care unit. Exclusion criteria were nurses and physicians who did not participate in a critical situation, a situation experienced as threatening the child’s well-being, in the child intensive care unit.

### 2.2. Data Collection

Semi-structured individual interviews were used as a data collection method. Participants showing interest were booked for interviews according to their wishes. Seven of the nine participants wanted to remain in the department to be able to interrupt the interview if the need arose. Before the interview began, both written and oral consent was obtained, and information was given on the right to suspend participation at any time. The interview guide originated from a literature review and was developed by experts in the field. After a pilot interview, the question guide was revised, with the question about the participants’ clinical experience with critical incidents added and one question reformulated for clarity. The pilot interview is not included in the study. Interviews span over September and October 2019, taking place in a separate room at the workplace or a booked room at another place in the hospital, similar to the phenomena’s natural environment. The interviews were recorded, and a semi-structured interview guide was followed to leave room for the participant to express themselves freely [22]. The authors let the informants themselves define what a critical situation means to them.

In summary, the participants described it as unplanned situations that cannot be foreseen, a situation where there are threats to the patient’s life, rapid deterioration in general condition and vital parameters, and failing organ systems. They also describe situations where quick action and more health care staff are required to stabilizing the child’s condition. The interviews were recorded on mobile phones in “airplane mode” to prevent interference. The interview guide was designed to start from more general questions and then moving on to more specific ones. Throughout the interview, follow-up questions were asked, such as: “What do you mean? Can you elaborate? Can you give me an example?”

Questions aimed at helping participants provide a sense of the phenomenon, following Polit & Beck [22]. All interviews started with the same question and varied between 25–40 min.

### 2.3. Data Analysis

Interview data were analyzed with a qualitative inductive content analysis based on Graneheim & Lundman [24]. The strength of content analysis in the present work is that the latent content, the underlying meaning of the content, can be analyzed. Another characteristic of qualitative content analysis giving strength to the work is that the method primarily focuses on the phenomenon and the context [24]. In step one, interviews were transcribed verbatim. Step two involved reading and listening through each transcript to ensure that the transcripts were correct. Step three was devoted to reading through the interviews several times to maintain a sense of the whole described by Graneheim and Lundman [24]. The material was read in its entirety several times during the process to understand the data. In step four of the analysis process continued, where units of meaning that corresponded to the purpose of the study were marked. In step five, the meaning units were entered into a document where the sentence units were condensed. According to Graneheim and Lundman [24], condensation means retaining the core of the content while shortening the text. In step six, the authors of this study analyzed what the informants said. Step seven meant comparing content for similarities and differences and then sort into categories and subcategories. In this step, three categories and two subcategories emerged that ultimately represent the results. A critical point in the analysis work, according to Graneheim and Lundman [24], to achieve credibility is to find suitable categories and subcategories for the meaning units.

### 2.4. Ethical Aspects

Consent to conduct the study was granted from the clinic’s head, and the Ethics Review Board approved an ethical permit in Stockholm, Dnr: 2017-1722-31-1. At the current PICU, the nursing care managers were contacted by email and given information about the study. After showing interest, the study was presented orally in a meeting, and written information was provided for the health care staff at the PICU. Information about the study was then emailed via work email to gain interested informants. At the time of the interview, information on the study was given orally and in a physical letter. An informed consent document was signed. Informed consent protects the participants’ autonomy and integrity. Before the interview began, it was again emphasized that participation was voluntary and could be aborted at any time. During the analysis process, all informants were encoded to ensure anonymity in the process. The basis of research ethics is to safeguard integrity, self-determination, and the equal value of all people affected [22], which has guided the authors throughout this study.

## 3. Results

The analysis conveyed that the medical needs weigh heavier and are more at the center than the holistic image of the child and his or her family. On the other hand, when the child’s life is threatened, there is a clear picture of including parents, but it is never clear when a critical situation turns into a life-threatening situation. Hindrance to let the parents be present could origin from fear of what could happen, both to oneself and the child. The resources in the team became the frame for how to handle the situation that arises. Only when a parent demands to be present does that demand exceed the decisions made by health professionals or when there was an obvious risk to the child’s life. Lack of resources within the team and fear of parents becoming a disturbance or a distraction are regarded as the primary causes of not letting the parents be present. Two categories and two subcategories, all connected to understand the phenomenon surrounding the health care staff’s perception of parents’ presence in critical situations, emerged through the analysis.

The category Fear describes how fears control health care staff and becomes an obstacle for parents to participate in critical situations. The subcategory Distribution of power highlights the health care staff’s power relationship with the parents. The health care staff, exercising their power, make their own decisions based on the child’s medical treatment. The subcategory, Resources in the team, highlights holistic care and its structure. The last category, Risk to the child’s life, describes how the health care staff agrees on parental presence when children are at risk of dying.

### 3.1. Fear

Parental attendance in a critical situation increases fear of failure and the fear of making a fool of oneself. In a critical situation involving parents, there is concern about medical procedures failing, risking the child’s life, which does not seem to exist to the same extent when the care team can act without parents as observers. The mere parent presence spreads nervousness in the team, which the team has not learned to handle, so parents instead are asked to leave the room. Unforeseen events where the health care staff experience a loss of control is a particularly stressful situation, where parents constitute a perceived disturbance and consequently are asked to leave.

*“In an emergency situation, it is not always the case that we control the situation ourselves. That we know what we, the underlying cause of the problem, what should be done, and if you are personally in such an uncertain situation, I think it is easier to sort of want to get rid of everything that can interfere so that you can have total concentration on the problem. And then it is easier than you want everything else to be removed. Including parents. (7)”*

In critical situations, the medical focus is the highest priority. Emphasis is on the importance of performing the medical procedure, and the risk of losing focus on the medical procedure bypasses the need for nursing care. Decision-making in the team is led by the physician, as the team believes that the physician has the most challenging task in this context. There is an underlying concern that parents will try to influence medical decisions by physically or verbally interfering and harming the child.

*“But I must also say that it could be that the parent takes focus from what we are doing. And since it is still the child who is the patient and the child that we are to work with, the parent must still come in, still in the second place. (3)”*

A person who has less experience tended to send parents out of the room, and a person with previous experience of participating parents was more likely to let parents participate. The more routine a situation is perceived as the more parents tend to be accepted in the room.

*“There are several factors that have been said, partly those who work that you feel more or less comfortable in your professional role to have someone who observes you when you work. I think it is easier to have, so to speak, an observer as parents or parents if you feel, that is, if you have worked for a while and you feel comfortable in your role. What you do; the more routine it is but thinks you do, the easier it is to have someone who observes you. If you do something that feels difficult, regardless, it can feel hard to have someone looking over your shoulder like… (8)”*

There is a perception that parents need protection from involvement in a critical situation, and with that notion, it is easier to ask parents to leave. The belief that the medical facts presented during a critical situation should frighten the parents creates a climate where the health care staff believe that they are doing the parents a favor by asking them to leave. If the situation arises suddenly and unprepared and the parents are in the room, sometimes staff does not reflect on their presence. These times parents often stay, forgotten, and not willingly leaving.

*“No. I do not think so. You just forget they are there. You focus on something else. Sometimes you can even forget them. (5)”*

In a situation with more time for preparation, parents are more likely to offer participation. Health care staff with good experience from parental involvement in the past encourage participation as they see its benefits, both as professionals and for the family.

*“It is so damn good when they see everything. But then you should be good too; then you should know your stuff. Not always beautiful if you do not, but you have to be very sure that you know what you are doing if you are always going to have them with you. That is my opinion. It’s… then you have nothing to hide behind. (1)”*

### 3.2. Distribution of Power

It is considered that the physician, who is to perform an advanced medical procedure, has priority in deciding who is allowed in the room. There is a consensus among the staff that the focus is medical practice. Nothing should interfere with this. Not even the child’s or parent’s need to be close to each other.

*“No, so I would not say that that, where I feel, know, I have the situations that have been, the PHYSICIAN has decided, there it is like the physician who decides because it is he who performs that moment. A procedure that it does as well as that the physician does. And they then decide how they personally want, how they want to do it. Most people want them to go out. (3)”*

Sometimes, the physician includes the team and the parents in creating a plan for carrying out procedures. The medical work is still in focus, but the parents can participate if desired.

*“…… I think it’s different in the group. There is no direct policy or consensus or agreement in the medical group in any case, but for me, the starting point is that the parents are with…. So, it’s an agreement I usually make with my parents. My starting point is that they are involved as well. And I do not think it is so for everyone maybe, but. (7)”*

If there is a fear that the parents may cause a disturbance, it is a decision made by the team or the physician who must perform a particular procedure to ask them to leave. If, on the other hand, parents are not satisfied with this decision and oppose it, they may sometimes be involved.

*“Then it is the physician who decides if parents are allowed to join. Then it is a pressured situation, and the physician is under pressure. And most people then say that they want them to wait outside. And I think I can understand that at that very moment. Then there has been someone, at some point, then there has been someone who, parents who have, as it were, tried to be involved. Still, and on those occasions, they have been involved. (3)”*

Attendance is also discussed based on the child’s age, where staff “feel” that a younger child has less need of parents as support, and therefore parents can be asked to leave the room. Health care staff have the power to decide whether parents are to be present or not if the parents themselves do not decide to take power over the situation and demand their participation.

### 3.3. Resources in the Team

Having a designated person taking care of the parents and enabling them to be present during critical situations was desirable as a health care professional’s primary task was to care for the child. If the staff felt it necessary, parents were often asked to wait outside or designated a specific location, informed that they would be fetched later. There is also the notion that parents could be involved to a greater extent with support. The absence of parents’ supporters was often used to excuse why parents were asked to leave. In such situations, parents are not offered to participate because the team has already decided otherwise.

*“Ehm but I think we should have it as far as possible and I think we often try to have parents and explain what you do and it is pretty good if you have someone who can explain and talk to the parents so you do not have to take and stand and lead the medical work or, as when you are in difficult things, that you should not have to pay attention to it without there being a separate person who can handle the parental contact. (6)”*

There is an unambiguity that the parents are involved to a greater extent when a designated person takes care of them. When resources are not available, the parents are asked to leave, even though the informants have never personally experienced parents as a direct disturbance. When the parents have been left alone in an emergency, staff describes parents as forgotten and not a disturbance.

*“No. I do not think so. You just forget they’re there. You focus on something else. Sometimes you can even forget them. (5)”*

### 3.4. Risk to the Child’s Life

When children are at risk of dying, there is consensus about the presence of parents. The parents are given space to be close to their child. Then focus shifts from the perspective of the profession and the child to focus on the parents’ possible future grieving process. There is a shared understanding that it will help the parents grieve if having seen what has happened and hearing how the care team has reasoned in such a situation. Staff describes relief because parents have seen that everything that could have been done has been done.

*“So, I think it is good for parents to see both what is being done, eh, and especially if it is so bad that it will not work. They actually get to see that you still work and that maybe they also sometimes need to hear us reasoning. (9)”*

In these situations, no doubt about one’s own competence is described, unlike other acute but not critical situations. The health care staff also does not express concern that parents will disturb care or other fears. There is no doubt that parents should participate in situations where there is a risk to the child’s life.

## 4. Discussion

As this study aimed to explore the health care staff’s perception of parents’ participation in critical situations, a qualitative design was used. The main result highlights that it is the health care staff who have the power to decide whether the parents can be present during critical situations or not. Fear is a driver in the decision of giving the parents the possibility to be present or not. When the focus shifts from saving the child to helping the child to a good death, the fear diminishes, and there is a joint agreement of including parents. According to Polit and Beck [22], a qualitative method is used to understand behavior, attitudes, and experiences. However, a qualitative method does not provide an absolute truth [22]. The method’s strength is to provide an in-depth analysis of a phenomenon that contributes to the nuance of reality. Correctly asked interview questions are essential for the informants to give a deep and exhaustive answer and be an excellent listener to ask good follow-up questions [22]. According to Polit and Beck [22], there is a risk that the informants are not completely honest during the interview situation so as not to expose their shortcomings. Therefore, various experiences and different professional categories were sought to get a comprehensive understanding of the phenomenon. Polit and Beck [22] describe that a select group with diversity is of great value as the patterns and structures that emerge in the results show core experiences.

In the present study results, the care of the critically ill child and his or her family has proved to be a complex task. There is knowledge from previous research about the importance of parents’ presence. As family members describe their experiences as a process, feeling unrepaired and lonely [25]. However, it is often overlooked for the child’s medical needs and sometimes also the fear of failure. In a Swedish study by Terp, Sjöström, and Strand [26], parents describe feelings of shame and guilt about leaving their child in the PICU. With the knowledge of this, the medical focus of the healthcare staff should be woven together with nursing care. Both physicians and nurses should treat patients with empathy, care, and respect, and in order to achieve child- and family-centered care, parents must be seen as a whole with their child. In the present study results, the health care staff describes that the primary focus in a critical situation is on the child’s medical condition. Parents are invited to participate if the focus can remain on the medical task. This type of behavior is also confirmed by an observational study conducted by Mattsson, Forsner, Castrén, and Arman [18], describing the nurses’ work primarily focused on the child’s medical condition, and that the child’s need for nursing care is not met.

Furthermore, Mattson et al. [22] describes that the medical focus is at the center and that the child’s needs come second. However, Arman and Rehnsfeldt [27] highlight the importance of treating the patient holistically to reduce suffering. By adopting child-centered care, the child as a whole becomes the focus [13]. Parents can interpret the child’s signals and bridge health care staff and children [11]. When parents do not participate, the child’s signals risk being misinterpreted or completely missed. Parents are an essential piece of the puzzle to achieve good quality in the care of the sick child, regardless of whether there is a critical situation or not. Twibell et al. [5] and Al Mutair [6] describes how health care staff with previous experience of parents’ participation are more positive about participation in similar situations later. This might indicate that staff needs to practice caring for parents in critical situations and training in medical tasks.

The result of the present study reveals a concern that the medical procedure in connection with a critical situation might be disturbed. This concern has been described in several previous studies and is a recurring phenomenon worldwide [3,4,8]. Concerns that parents should physically get involved are shared globally. However, this fear is based on anecdotes [2]. Less experienced nurses might feel vulnerable when alone with parents in the ward and need more support, especially when feeling confronted by parents about working methods and skills [9]. The interviewee’s concern about being questioned by parents was described regardless of experience in the field, indicating that years in the occupation are of little importance, but rather previous experiences. The Convention on the Rights of the Child declares that children have the right to security in health care, which means that the child has the right to have a parent present whenever he or she wants [11]. Parents expressed a need for support without being judged or scrutinized, choosing to be present or absent. When parents have not been able to stay with their children, the parents have denied the situation and denied the reality. With the proper support, the parents experienced less anxiety, regardless of whether they stayed or not [26]. As there are no clear guidelines regarding the presence of parents in critical situations, only a few countries apply guidelines [2]. When guidelines are lacking, and there is no developed working method, the power is left unconsciously to the health care staff.

Decisions are made believing that the team does the best for the parents, sometimes on ill-considered grounds. There is a perceived image that parents should be both afraid and experience discomfort from witnessing healthcare interventions. Which makes health care staff exclude them, and at the same time, express an image of not wanting to be observed and examined by parents during a critical situation. The National Board of Health and Welfare highlights the importance of strengthening the patient perspective with guidelines [15]. A suggestion is that staying with the child in the critical situation should be discussed at admittance to the PICU. Ekman et al. [10] describe that the concept of power characterizes the relationship of dependence between health care staff and care recipients. As a caregiver, you have both knowledge and powers, and as a patient, you are in a position of dependence. It is essential to preserve the patient’s dignity, integrity, and autonomy in such a power relationship. According to the National Board of Health and Welfare [28], healthcare must inform the patient or their guardians about treatment to strengthen and clarify their self-determination and participation. Family-centered nursing is based on a non-hierarchical relationship characterized by humility and reciprocity to the patient and their parents [29]. If there is no parental support, the staff often consider that parents might constitute a potential disruption, and they must wait outside.

The lack of staff resources, space, and time are stated as a reason to exclude parents is described in several studies [3,4]. Parents of children in intensive care need to feel invited by the health care staff [30]. Things as being offered a place to sit next to the bed were considered an inviting gesture, and when the health care staff showed where it was safe for the parents to sit, a family space was created, an anchor point. However, when the child’s life was at risk, the presence of parents is considered self-evident. An understanding of a facilitated grieving process is expressed by the informants, which is also supported by previous research [21,29,30,31,32]. The informants expressed relief that parents have heard what was said and that parents have seen that the staff did everything they could [3,32].

## 5. Conclusions

The present study has identified that power distribution in critical situations is shifted to the health care staff and leaves children and parents in neglect. Given this, an educational intervention to practice critical incidents with parents present is of utmost importance to increase parental participation in such situations. It should be a natural way of working, including parents in the child’s care, and parents should have a designated place to stay in critical situations before the situation. Moreover, decreasing the anxiety health care staff might experience when parents are present.
